# A Mozambican marine protected area provides important habitat for vulnerable pelagic sharks

**DOI:** 10.1038/s41598-023-32407-9

**Published:** 2023-04-20

**Authors:** Calum J. G. Murie, Mario Lebrato, Andrew Lawrence, James Brown, Livia Gavard, Karen R. Bowles, Mauro G. Jije, Matt Dicken, Simon P. Oliver

**Affiliations:** 1 Department of Biological Sciences, University of Chester, Chester, CH1 4BJ UK; 2 Underwater Africa, Tofo, Inhambane, Mozambique; 3 Bazaruto Centre for Scientific Studies (BCSS), Bazaruto Archipelago, Inhambane, Mozambique; 4 KwaZulu Natal Sharks Board, Umhlanga Rocks, 4320 South Africa; 5 School of Biological and Marine Sciences, University of Plymouth, Plymouth, PL4 8AA UK; 6 The Thresher Shark Research and Conservation Project, Malapascua Island, Cebu, The Philippines

**Keywords:** Animal migration, Behavioural ecology, Conservation biology

## Abstract

Pelagic sharks play key roles in marine ecosystems, but are increasingly threatened by human extraction, habitat degradation and mismanagement. We investigated the use of protected and unprotected coastal habitats by bull (*Carcharhinus leucas*) and oceanic blacktip (*Carcharhinus limbatus*) sharks in southern Mozambique. Five INNOVASEA VR2W-69 kHz acoustic receivers were positioned in the Bazaruto Archipelago National Park (BANP) as well as one to the south of the park’s boundaries. Seven receivers were also deployed 250 km south in the Inhambane estuary and on reef sites off Praia de Tofo. Twelve bull, and six oceanic blacktip sharks, were fitted with INNOVASEA V16 acoustic tags, which generated 933 detections of bull and 12,381 detections of oceanic blacktip sharks over a period of 1391 days. A generalised additive model was used to estimate the effects of seven spatiotemporal and environmental parameters on the frequency of each species’ detections. In general, calculated residency indices were highest around the locations monitored in the BANP and one unprotected location off Tofo. Both species were more abundant across the monitored sites, during the summer when water temperatures were ~ 27 °C, when the moon was < 50% illuminated, and when the tide was rising. Detections coincided with each species’ reproductive season indicating that both species may be reproductively active in the BANP region. Oceanic blacktip sharks were largely resident and so fisheries management may significantly benefit their population(s) around certain reef habitats in the BANP. The low residency and seasonal detections of bull sharks indicates that they may be transient and so effective conservation may require coordination between regional fisheries managers.

## Introduction

Large shark species can exert strong influences on the structure and function of the ecosystems that they inhabit, but many of their life history characteristics, such as slow growth, late maturity, and low fecundity make them susceptible to overexploitation^1,2^. The movements and habitat use of many coastal shark species have yet to be described since their transboundary movements make tracking investigations inherently challenging^1^. However, studies on the spatial ecology of large oceanic sharks have revealed social, foraging, and reproductive behaviours, and have helped to identify important habitats^1,3–5^. Understanding patterns in the spatial ecology of sharks is an important precursor to being able to manage and protect them effectively^3,4^. Therefore, knowledge of an oceanic shark’s spatial ecology, and especially their use of coastal habitats, has become an important priority for managers working to protect oceanic shark populations across the globe^1,6^.

Effectively managing oceanic sharks in marine protected areas (MPAs) can significantly benefit their populations^6,7^. Regulated fishing activities, and enforced bans on destructive practices (e.g., long-lining, gillnetting), can create more productive and biodiverse marine habitats that support fish biomass, and provide sharks with abundant prey^6,8^. The extent to which oceanic sharks benefit from MPA’s is debated, but it has been suggested that protected habitats where anthropogenic disturbance and extractive pressures are managed, can function as refugia and undisturbed areas for reproducing and socialising^9^. Acoustic biotelemetry has proven effective when investigating how oceanic sharks use inshore habitats and how their movements interact with MPA boundaries^9–11^. Understanding how sharks make use of habitats both inside and outside of MPAs can aid in the development of regional management strategies and facilitate the implementation of seasonal fisheries regulations, habitat protection, and refined/expanded MPA boundaries that better reflect patterns in their habitat use^4^.

Globally vulnerable bull (*Carcharhinus leucas*) and oceanic blacktip (*Carcharhinus limbatus*) sharks are large, apex marine predators that are regularly caught by coastal fisheries and have been implicated in near-shore shark attacks^12–14^. Acoustic studies have identified that spatiotemporal and environmental variables such as the season, time of day, water temperature (reported preference ~ 25 °C), tidal flow, and lunar cycle, influence their abundance and use of coastal habitats^1,10,14,15^, (including MPAs) where they feed, socialise, and reproduce^8,11,16,17^. Knowledge of the fine and large-scale movements of numerous coastal shark species has enabled regional managers to refine spatial protection strategies and strengthen regional fisheries policy for the benefit of associated populations^6–8,11^. In this study we investigate patterns in the coastal habitat use of bull and oceanic blacktip sharks across ~ 400 km of coastline in southern Mozambique to address the following hypotheses: (1) *C. leucas* and *C. limbatus* show patterns of residence at and around specific locations; (2) their detections around the monitored locations differ on a seasonal basis; and (3) patterns in their use of the monitored habitats are driven (at least in part) by environmental factors. Results are discussed in the context of regional shark conservation.

## Results

### Detections

**Figure 1:**
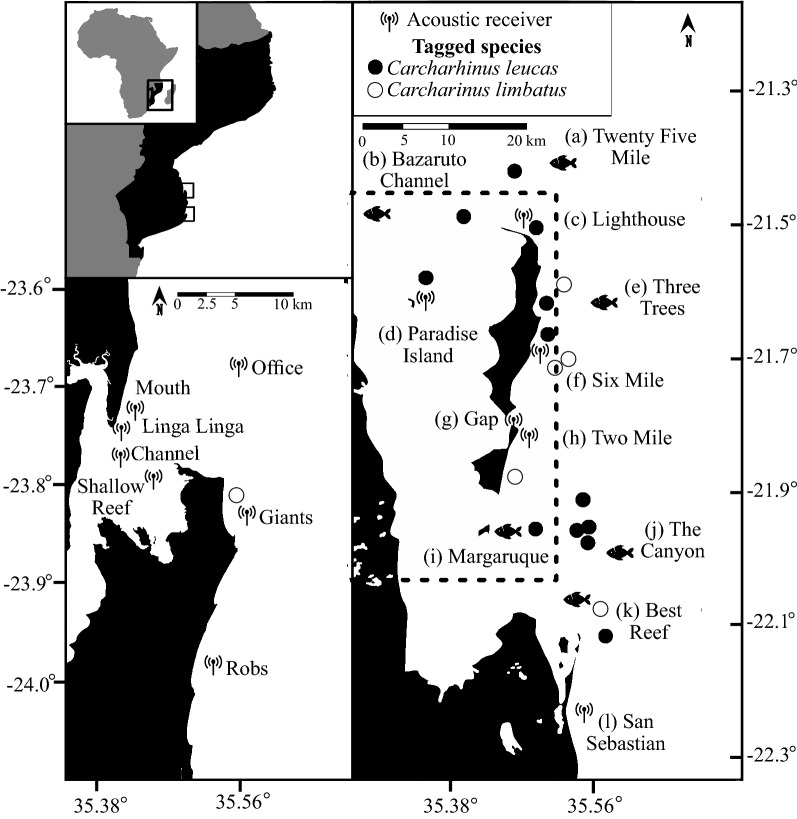
Location of the deployment of the acoustic transmitters and receivers () in the Inhambane province of Mozambique. The () symbol represents locations where acoustic tags were deployed but no receivers were stationed. Locations where *Carcharhinus leucas* were tagged are characterised by a dot (●) and *Carcharhinus limbatus* by an empty circle (○). Labels (a … r) refer to Table 2. The dotted line denotes the boundaries of the Bazaruto Archipelago National Park. The axes represent latitudinal and longitudinal positions.

18 sharks were fitted with INNOVASEA V16 8L acoustic transmitters (12 bull sharks (*C. leucas*) and six oceanic blacktips (*C. limbatus*)) (Table 1). One oceanic blacktip was fitted with a transmitter off Praia de Tofo and all of the other sharks were fitted with transmitters at locations both inside and outside of the BANP perimeter (Fig. 1). Bull sharks had a mean (± SE) pre caudal length of 190.5 ± 12.22 cm (minimum: 123 cm, maximum 286 cm), a fork length of 217 ± 11.92 cm (minimum: 169 cm, maximum 311 cm), and a mean total length of 252.08 ± 15.11 cm (minimum: 186 cm, maximum 376 cm). Oceanic blacktip sharks had a mean (± SE) pre caudal length of 140.17 ± 10.9 cm (minimum: 94 cm, maximum 163 cm), a fork length of 172.17 ± 14.75 cm (minimum: 102 cm, maximum 181 cm), and a mean total length of 197 ± 15.75 cm (minimum: 123 cm, maximum 198 cm). Of the 18 sharks that we tagged, 9 (5 bull sharks and 4 oceanic blacktip sharks) were detected by the deployed receivers following the sharks’ release. Bull sharks were monitored for a mean (± SE) total of 676.25 ± 31.92 days at liberty (minimum: 536 days, maximum: 892 days) whilst oceanic blacktip sharks were monitored for a mean total of 852.67 ± 91.99 days at liberty (minimum: 606, maximum 1174 days) (Table 1). Acoustic receivers recorded 13,321 total detections of sharks, including 933 detections (7.01% of total) from the tagged bull sharks (n = 12) and 12,388 from the tagged oceanic blacktip sharks (n = 6; 92.99% of total) (Table 1). For bull sharks, 10 false detections were removed prior to analyses and for oceanic blacktips 138 detections were removed.

**Table 1:** Details of the tagged sharks including their Latin Name, ID code, Sex, and length of the tagged sharks, tagging location and date, monitoring duration, and total, unfiltered, detections for each of the acoustic receivers. The asterisk denotes a pregnant shark in late stage pregnancy at time of tagging.

ID	Latin name	Shark lengths (cm)			Sex	Location tagged	Total days monitored	Total detections
		Caudal	Fork	Total				
1	*C. leucas*	123	169	186	Female	(b) Bazaruto Channel	–	0
2	*C. leucas*	172	214	242	Female	(c) Lighthouse	868	869
3	*C. leucas*	185	202	218	Female	(j) Canyon	–	0
4	*C. leucas*	187	209	227	Male	(j) Canyon	–	0
5	*C. leucas*	194	219	230	Male	(j) Canyon	–	0
6	*C. leucas*	253	276	305	Female	(k) Best Reef	687	24
7	*C. leucas*	187	198	261	Female	(e) Three Trees	–	0
8	*C. leucas*	286	311	376	Female*	(i) Magarruque	633	1
9	*C. leucas*	165	179	219	Female	(a) Twenty-Five Mile	622	7
10	*C. leucas*	207	247	303	Female	(j) Canyon	604	32
11	*C. leucas*	168	202	243	Female	(b) Bazaruto Channel	–	0
12	*C. leucas*	159	187	215	Female	(f) Six Mile	–	0
13	*C. limbatus*	144	206	230	Female	(r) Giants	1174	3408
14	*C. limbatus*	126	186	211	Female	(e) Three Trees	1138	406
15	*C. limbatus*	163	181	198	Female	(f) Six Mile	814	3554
16	*C. limbatus*	165	186	223	Female	(f) Six Mile	–	0
17	*C. limbatus*	149	172	197	Female	(k) Best Reef	668	5020
18	*C. limbatus*	94	102	123	Female	(h) Two Mile	–	0

**Table 2:** Residency indices for each acoustic receiver location that recorded sharks tagged with acoustic transmitters. Only sharks with one recorded detection at least one receiver site are included, otherwise the values are zero. The letter K denotes the Kessel method (Distinct number of days detected at the location/distinct number of days detected at any location) whilst the letter TI indicates the Time Interval method (600 s (see “Methods”)) (Distinct number of time intervals detected at this location / distinct number of time intervals detected at any location). The Index headings refers to residency indexes calculated using the Kessel (K) and Time Interval (TI) methods. The Days Detected heading refers to the number of days and/or proportion of the day that sharks were detected around a particular receiver. Labels (c, f, g, h, l, r) refer to locations illustrated in Fig. 1.

ID	Species	(c) Lighthouse				(f) Six Mile				(g) Gap				(h) Two Mile				(l) San Sebastian				(r) Giants				Total days detected	
		Index		Days detected		Index		Days detected		Index		Days detected		Index		Days detected		Index		Days detected		Index		Days detected			
		K	TI	K	TI	K	TI	K	TI	K	TI	K	TI	K	TI	K	TI	K	TI	K	TI	K	TI	K	TI	K	TI
2	*C. leucas*	–	–	–	–	–	–	–	–	–	–	–	–	–	–	–	–	–	–	–	–	1	1	6	2.5	6	2.5
6	*C. leucas*	0.22	0.22	2	0.5	0.67	0.67	6	1.5	–	–	–	–	–	–	–	–	0.11	0.11	1	0.25	–	–	–	–	9	2.25
8	*C. leucas*	–	–	–	–	1	1	1	0.25	–	–	–	–	–	–	–	–	–	–	–	–	–	–	–	–	1	0.25
9	*C. leucas*	1	1	4	1	–	–	–	–	–	–	–	–	–	–	–	–	–	–	–	–	–	–	–	–	4	1
10	*C. leucas*	0.22	0.2	2	0.5	0.78	0.8	7	2	–	–	–	–	–	–	–	–	–	–	–	–	–	–	–	–	9	2.5
***C. leucas***		**0.28**	**0.23**	**8**	**2**	**0.48**	**0.44**	**14**	**3.75**	**0**	**0**	**0**	**0**	**0**	**0**	**0**	**0**	**0.03**	**0.03**	**1**	**0.25**	**0.21**	**0.29**	**6**	**2.5**	**29**	**8.5**
13	*C. limbatus*	–	–	–	–	–	–	–	–	–	–	–	–	–	–	–	–	–	–	–	–	1	1	225	125.5	225	125.5
14	*C. limbatus*	0.97	0.98	76	27.25	0.01	0.01	1	0.25	–	–	–	–	0.01	0.01	1	0.25	–	–	–	–	–	–	–	–	78	27.75
15	*C. limbatus*	0.24	0.12	62	24.25	0.2	0.14	18	5	0.77	0.84	68	31.25	0.03	0.02	3	0.75	–	–	–	–	–	–	–	–	151	61.25
17	*C. limbatus*	–	–	–	–	–	–	–	–	0.45	0.46	5	4	–	–	–	–	0.55	0.54	6	4.75	–	–	–	–	11	8.75
***C. limbatus***		**0.43**	**0.27**	**108**	**51.5**	**0.05**	**0.03**	**19**	**5.25**	**0.18**	**0.18**	**73**	**35.25**	**0.01**	**0.01**	**4**	**1**	**0.06**	**0.02**	**6**	**4.75**	**0.56**	**0.64**	**225**	**125.5**	**435**	**223.3**
***Carcharhinus spp.***		**0.19**	**0.13**	**116**	**27.25**	**0.08**	**0.04**	**33**	**9**	**0.17**	**0.17**	**73**	**35.25**	**0.01**	**0.004**	**4**	**1**	**0.02**	**0.02**	**7**	**5**	**0.54**	**0.62**	**231**	**128**	**464**	**231.8**

Significant values are in [bold].

### Spatial–temporal influences

**Figure 2:**
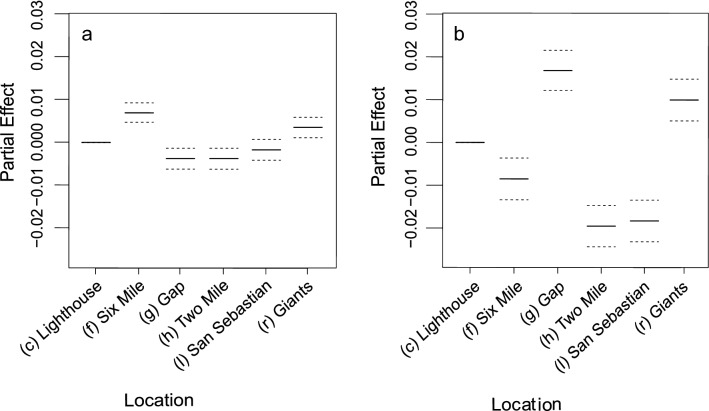
Partial effects of receiver location (**a**, **b**) on recorded detection events for *Carcharhinus leucas* (**a**; n = 12) and *Carcharhinus limbatus* (**b**; n = 6) sharks. Location labels (c, f, g, h, l, r) reference Table 2 and refer to the locations that acoustic receivers were deployed across the Inhambane province of southern Mozambique. Solid lines refer to the partial effect of each receiver location on the frequency of detection events and dotted lines represent the confidence intervals.

**Figure 3:**
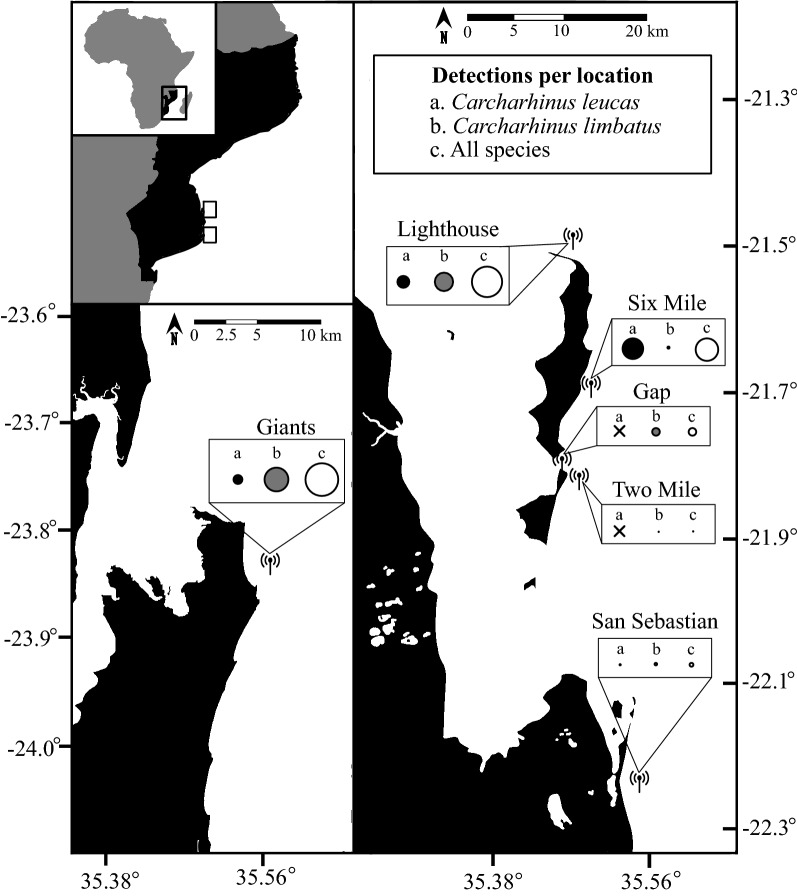
The number of detections recorded for tagged bull, *Carcharhinus leucas,* and oceanic blacktip, *Carcharhinus limbatus,* sharks. The size of the circle describes the number of the detections that were recorded at that location. White circles denote both species detections, grey circles characterise detections of oceanic blacktip sharks, and black circles denote detections of bull sharks. The radio antennas describe receiver locations that made at least one detection of a tagged shark.

Inside of the BANP MPA bull sharks were most likely to be detected by the Six Mile (f) and Giants (r) receivers, followed by the Lighthouse (c), and San Sebastian receivers (l) (F(4.813) = 7.903, *p* < 0.001), and their residency scores were calculated to be greatest around the Six Mile (f) and Lighthouse (c) receivers (Figs. 2, 3; Table 2). Oceanic blacktip sharks were most likely to be detected by the Gap (g) receiver, Six Mile (f) and (in lower numbers) around the Two Mile (h) receiver (F(5.487) = 9.876, *p* < 0.001). Oceanic blacktip residency scores were highest at the Giants (r), Gap (g), and Lighthouse (c) receivers (Figs. 2, 3; Table 2). Of the unprotected sites, detection events were only recorded around the Giants (r) and San Sebastian (l) receivers (Figs. 2, 3; Table 2). For bull sharks, similar high rates of detection were recorded for the Giants (r) receiver, which is unprotected, and the Six Mile (f) and Lighthouse (c) receivers, which are protected. For oceanic blacktip sharks, similar high rates of detections were recorded around the Giants (r) and Gap (g) (located at the centre of the BANP MPA) receivers. A small number of detections, and low residence scores, were also recorded for bull sharks at the San Sebastian (l) receiver location, off the southern border of the BANP MPA (Figs. 2, 3; Table 2).

**Figure 4:**
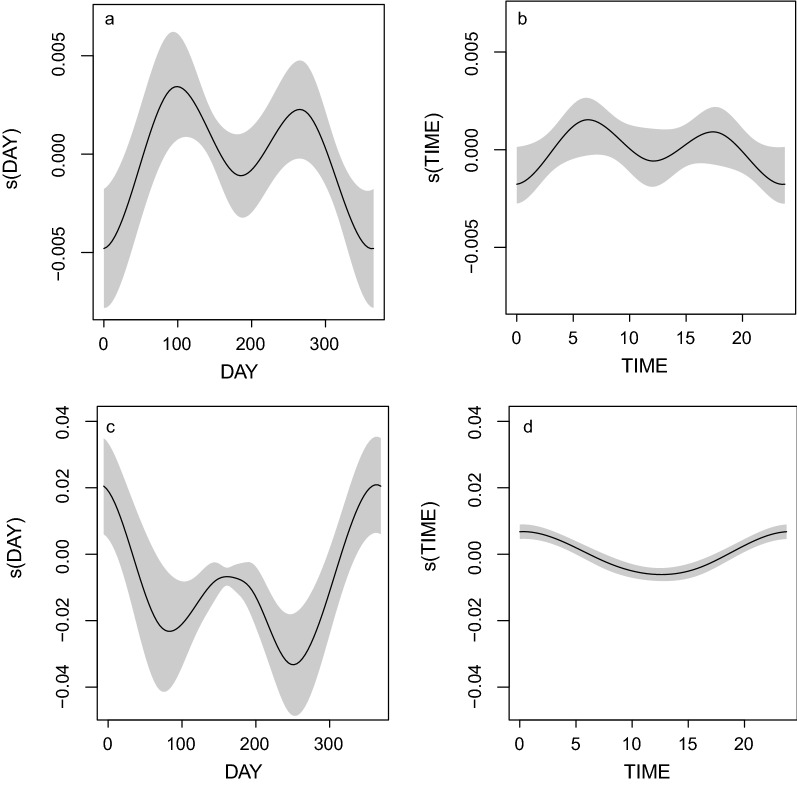
Generalized additive model (GAM) plots showing the partial effects of day of the year and time of day on the frequency of *Carcharhinus leucas* (**a**, **b**) and *Carcharhinus limbatus* (**c**, **d**) shark detection events at receiver locations throughout the Inhambane province of southern Mozambique. Shaded areas indicate the 95% confidence intervals.

Bull shark detections were most frequent during March and October (F(2.901) = 6.053, *p* < 0.001; Fig. 4a), rare around December and January and most commonly occured during the early morning (06:00–08:00) and late afternoon (17:00–19:00) (F(2.688) = 3.403, *p* = 0.019; Fig. 4b). Oceanic blacktip shark detections peaked between December and Janurary (df = 6.886, F = 13.707, *p* < 0.001; Fig. 4c) and occurred most often at night (df = 1.897, F = 19.810, *p* < 0.001; Fig. 4d).

### Environmental influences

**Figure 5:**
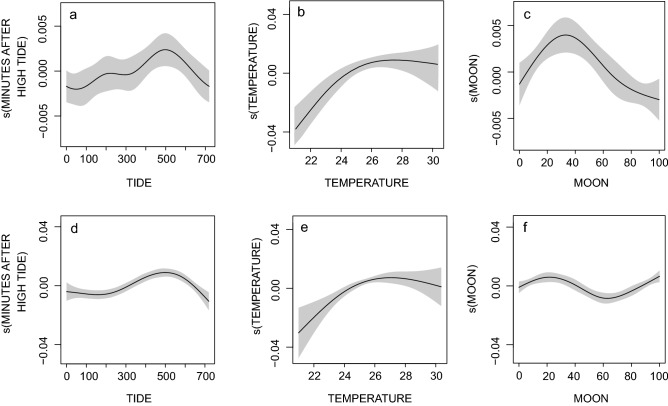
Generalized additive model (GAM) plots showing the partial effects of the minute after high tide, water temperature and the moon illumination on the frequency of bull (**a**–**c**) and oceanic blacktip (**d**–**f**) shark detections events at receiver locations throughout the Inhambane province of southern Mozambique. Shaded areas indicate the 95% confidence intervals.

Bull (df = 2.869 F = 4.008.403, *p* = 0.029) and oceanic blacktip shark (df = 4.040, F = 11.497, *p* < 0.001) detections were greatest during rising tides and peaked when water temperatures were between 26 and 28 °C (df = 1.369, F = 3.046, *p* = 0.028, df = 2.496, F = 7.703, *p* < 0.001; Fig. 5). Bull shark detections occurred most frequently when the moon was 20–40% illuminated (df = 2.784, F = 7.871, *p* < 0.001; Fig. 5c). Oceanic blacktips were most frequently detected when the moon was 20–40% or, 100% illuminated (df = 4.013, F = 7.903, *p* < 0.001; Fig. 5f).

## Discussion

This study represents the first investigation into the spatial ecology of bull (*C. leucas*) and oceanic blacktip (*C. limbatus*) sharks in Mozambican waters and suggests that waters in the Inhambane province and the Bazaruto Archipelago National Park (BANP) are important habitats for these species. Associated knowledge will be of interest to managers working to protect sharks in the BANP, and throughout the Western Indian Ocean (WIO).

All but four of the tags were deployed around the perimeter of the BANP, and most of the detections were recorded at reef sites inside the BANP. More than 90% of the sharks that we encountered and tagged in the BANP were female and of a size widely accepted as sexually mature^18^. A bull shark that we tagged in shallow waters inside of the MPA’s boundary (shark n° 8, ~ 376 cm total length, tagged on 8/12/2019) was also assessed to be in a late stage of pregnancy. It has been suggested that bull and oceanic blacktip sharks are reproductively philopatric and some MPA’s have been shown to encompass habitat where the sharks breed, socialise, and forage^7,19,20^. If bull and oceanic blacktip shark migrations into the BANP form part of their reproductive strategies, then targeted management of their populations both inside the BANP and in the surrounding waters could have significant benefits for their regional conservation by protecting female sharks while they gestate^11,18,21^. The lack of recorded detections in the Inhambane estuary suggests that sharks do not regularly enter the system^1^. This could be due to several factors, including a lack of reliable food sources, suitable breeding habitat or, increased disturbance^15,21^.

Most of the detections of the tagged sharks occurred when the water was warm (~ 27 °C). Water temperature is closely affiliated with physiological processes in sharks that promote metabolism, growth, respiration, and reproduction^15,22^. Bull and oceanic blacktip sharks are known to prefer water temperatures between 26 and 28 °C in the USA and Australia^5,15,22^, and it has been suggested that they move through these temperatures to thermoregulate which can trigger reproductive responses in these locations^15,22^.

Most of our bull shark detections were recorded in October and March, whilst detections of oceanic blacktip sharks were most frequent during the austral summer, which is consistent with most records of their reproductive behaviours in the southern hemisphere^23–25^. The plotted distributions of both species’ detections were near reflections of one another, with opposing peaks and throughs (Fig. 5), which may indicate that seasonal drivers are uniquely influencing each species or, since these sharks predate on one another, that one species avoids the monitored locations when the other becomes abundant^1,20^. The sexual maturity of the female sharks that we tagged, and the increased indices of their residency during months when the water was warm may indicate that the sharks visit the study area to gestate, although the large size of the captured individuals, and the presence of mating scars on all tagged females, may indicate they also court and mate in the region^23,25^. Since both shark species demonstrated strong commitments to specific sites (particularly to those in the BANP) where they may be reproductively active, managers should consider enhancing spatiotemporal protection of these areas by banning the extraction of sharks around all these sites. Particular attention should be given to the unprotected Giants site, formalising their protection inside the BANP and, widening the existing ‘Zones of Total Protection’ in the BANP to encompass their high-use habitats^22^.

Bull sharks were detected less frequently than oceanic blacktip sharks and body size can be a strong predictor for a species’ home range^3^. Bull sharks grow to a total length of ~ 3.6 m, which is larger than the ~ 2.4 m maximum expected growth for oceanic blacktips, and bull sharks have been shown to undertake substantial migrations to transition between seasonal foraging and/or breeding grounds^18,26^. Migratory behaviour is thought to be particularly pronounced in female bull sharks that are sexually mature, and migrations have been linked to their reproductive strategy^11,18,26,27^. Whilst previous research has indicated that in South Africa bull sharks have relatively small home ranges^23^, our data suggest that they have periods of residency in southern Mozambique that may be interspersed with substantial (> 500 km) migrations^18^. The residency of oceanic blacktip sharks is thought to increase around inshore tidal areas, large bays, and estuaries^21,28^, which is consistent with the physical characteristics of the BANP. The relatively high residency scores that we calculated for oceanic blacktip sharks in the BANP MPA may indicate that these habitats are important to their populations. Fisheries moratoriums on Carcharhinids and the management of the coral reefs that surround the receiver locations, particularly around the Giants, Six Mile, Lighthouse, and Gap locations, would benefit their regional populations^11,18^.

Detections of both shark species varied with the time of day, moon illumination, and the state of the tide. Oceanic sharks are known to use tidal flows to access specific habitats, improve the efficacy of ram ventilation, and facilitate their movements^29,30^. Our detections of both bull and oceanic blacktip sharks, around the monitored inshore reefs, were most frequent during rising tides. By swimming with the tides, the sharks may reduce some of the bioenergetic costs associated with movement^28,31^. In southern Mozambique bull and oceanic blacktip sharks principally forage on large teleost species (e.g., *Caranx* spp.) which are known to congregate around inshore reef systems^14,18,24^. The detections that we recorded may indicate that both sharks forage on inshore reefs during rising tides^14,18,24^.

Bull sharks were most frequently detected in the early morning and late afternoon when the moon was < 50% illuminated whilst oceanic blacktip sharks were detected most frequently at night when the moon was full or < 50% illuminated. Since Carcharhinids are generally ambush predators, they make use of low light levels to enhance their foraging success^14,18^. The nocturnal detections of oceanic blacktip sharks may indicate that this species exhibits a nocturnal foraging pattern, whilst the bull shark detection patterns may indicate that they visit these sites to forage during the twilight hours^14^. During tagging, large aggregations of bull and oceanic blacktip sharks were routinely observed close to the surface. The sharks’ appeared to forage on bonito (*Euthynnus affinis*), skipjack (*Katsuwonus pelamis*) and yellowfin tuna (*Thunnus albacares*) shoals when they surfaced, and this was observed along depth contours spanning from 20/30 m to 300 m depth suggesting that the sharks may be following the shoals. Since tuna migrate south into the studied region in November before moving northwards in March, the extent that the sharks follow them and subsequently transition out of the BANP region remains unknown^32^. During our observations a bull shark was detected in the BANP MPA, before being detected on a reef site directly off Praia de Tofo, indicating that at least some individuals move away from the MPA^18^. Future studies should investigate the extent to which bull and/or oceanic blacktip sharks’ range outside of the BANP’s waters with a view to estimate the degree to which they are threatened by the region’s abundant commercial and artisanal fishers^3,22^.

Many large, wide-ranging, oceanic shark species regularly return to specific inshore regions or sites, including MPAs, that support aspects of their life history strategies^3^. Understanding patterns in their spatial ecology can help to refine shark management strategies and yield conservation benefits^4^. Our findings indicate that the waters off the BANP represent important habitat for both bull and oceanic blacktip sharks. The recorded seasonal distribution of detections supports our assertion that the waters off the BANP function as seasonal breeding grounds^18^. Oceanic blacktip sharks appear to be largely resident around the monitored locations. Improved in situ fisheries regulations, and the protection of high use habitats, would promote protection for the region’s shark populations^3^. Since bull sharks appeared to be transient the BANP authorities should coordinate with regional fisheries managers to establish and protect common corridors of movement to sustain their abundance within the BANP^22^. Our findings indicate that both species may be reproductively active in the region, which should be the focus of future work since, the overexploitation of sexually mature/pregnant sharks could prove detrimental to the sustainability of populations in Mozambique and the Western Indian Ocean (WIO) region.

## Methods

This work complied with the University of Chester’s research ethics framework under 1669/20/CM/BS (granted to CM and SO. Fieldwork was undertaken in collaboration with the Underwater Africa Foundation (No: 101351548) and the Bazaruto Centre for Scientific Studies (BCSS) (No: 101218511) and was undertaken by permit (the Department of Conservation 04/GDG/ANAC/MTA/2020). All the methods complied with relevant guidelines and regulations.

### Location

The coastline of southern Mozambique hosts an array of tropical and subtropical marine ecosystems that support many elasmobranch species^33^. The waters off the Bazaruto Archipelago (− 21.79073, 35.47140) in the Inhambane province were designated as a Marine Reserve in 1971 and consolidated into a park in 2001 (Bazaruto Archipelago National Park—BANP). Deep waters (> 800 m) are located 2–3 km from the archipelago and connect to the islands via canyons, deep-water channels, and tidal inlets (Fig. 1). A series of barrier and pinnacle reefs surround the region and local fishermen report that these reefs support large aggregations of sharks. The archipelago’s inshore water movements are dominated by the tide, and support seagrass meadows and mangrove forests that function as nursery grounds for a variety of fish and elasmobranch species^33^.

Praia de Tofo, which is a fishing village and tourism hotspot, lies 200 km south of the BANP (− 23.85788, 35.54044). Its coastline supports similar ecosystems to those of the BANP with a large, tidal, inshore estuary (the Inhambane estuary), and a series of reefs bordering the oceanic drop off. The Inhambane estuary/bay is a large tidal lagoon with two seasonal freshwater inputs (Fig. 1). A deep-water (~ 28 m) channel runs the length of the bay and splits off into a series of shallower (< 10 m) channels that are surrounded by intertidal sand banks and mangrove forests (Fig. 1). The reefs fringing the coast of Praia de Tofo are easily accessed by megafauna, but its waters are unprotected (unlike the BANP), and sharks have been targeted by fishers for many years^34^.

### Acoustic monitoring

Thirteen omni-directional INNOVASEA VR2W acoustic receivers (Amirix Systems Inc., Nova Scotia, Canada) were deployed at protected and unprotected habitats throughout the Inhambane province of southern Mozambique. Five acoustic receivers were deployed inside the BANP’s boundaries to investigate if sharks move through or reside within the park’s jurisdictional waters, and one was positioned outside to monitor shark movements around the BANP’s southern border. A receiver was deployed on a reef at the midpoint of Bazaruto island [(f) Six Mile] to determine if sharks visit or transition along the island’s seaward coast. A second BANP receiver was positioned on the sandy bottom off Santa Carolina [(d) Paradise Island], on the leeward side of Bazaruto island, to examine if sharks move inshore. A third receiver was positioned in the channel that splits Bazaruto and Benguerra island to examine the level of shark residence around this location to estimate if sharks use it to transition between the BANP’s inshore and coastal environment [(g) Gap]. The fourth BANP receiver was positioned on a reef off the east coast of Benguerra island [(h) Two Mile] to determine if sharks’ transition or reside along the island’s seaward coast. The final BANP receiver was positioned on a reef site off the northern tip of Bazaruto [(c) Lighthouse] to investigate potential shark residence around the BANP’s northern perimeter (Fig. 1). The sixth and final receiver [(l) San Sebastian] was deployed on a reef site off the San Sebastian peninsula (20 km south of the BANP border) to estimate if sharks reside around an unprotected group of reefs close to the BANP perimeter (Fig. 1).

Seven receivers were stationed off Praia de Tofo to examine shark movements towards, and potential residence around, unprotected locations (Fig. 1). Since mangrove systems in estuarine environments can function as Carcharhinid nurseries^19^ four receivers were positioned in the Inhambane estuary/bay, inshore of Praia de Tofo. A receiver was positioned in the estuary’s main access point and dredged channel to detect any sharks entering and exiting the estuary [(n) Mouth]. The second was deployed in the entrance of a large mangrove channel [(o) Linga Linga], the third in the estuary’ main channel [(p) Channel] and the fourth in the shallows to the southeast of the estuary [(q) Shallow Reef]. To estimate shark residence around open ocean reef sites and investigate potential connectivity to similar inshore reef sites in the BANP, three receivers were positioned at reef systems to the North, South and directly off Praia de Tofo.

Receivers were tethered to a 1.5 m meter metal pole that was recessed into a 50 kg (dry weight) 3D-printed concrete and sand block (total height from benthos = 1.6 m). Range tests^35^ were performed to ensure that the receivers were functional prior to deployment. Receivers were deployed by two divers using SCUBA gear in 6–28 m depths and recovered every six months to change the batteries and download the data (Fig. 1). Testing revealed that receivers positioned in the BANP had a mean detection range of 606.5 m ± 32.29 m. Receivers in the Inhambane estuary had a mean detection range of 574.32 m ± 46.31 m, and receivers off Praia de Tofo tested at 692.33 m ± 22 m (Fig. 1).

To track individual bull and oceanic blacktip sharks, INNOVASEA V16 8L acoustic tags (Amirix Systems Inc., Nova Scotia, Canada) set with a randomised nominal delay of 120–180 s (estimated battery life: 3300 days) were sanitised with 100% alcohol and surgically implanted into the peritoneal cavity of captured sharks. Sharks were captured with a 200 lbs rod and reel using 18/0–22/0 circle hooks on tuna baits and brought alongside a research vessel. A tail rope (wool coated nylon ~ 5 cm diameter) was attached around the caudal peduncle, and they were carefully rotated until the ventral surface was exposed. Transmitters were inserted through a 2 cm incision which was disinfected and sealed with 2 independent sutures of braided silk^5^. During the tagging process, sharks were sexed, and measured^36^. All the sharks were assessed to be healthy after the procedure and swam away without assistance.

Environmental and temporal data including the date, time of the day (measured as minutes from 00:00 h), tidal state (measured as minutes after the high tide), tidal range (cm), and moon illumination (% of the full moon—tide and lunar data downloaded from: www.tides4fishing.com) were recorded. The water temperature (measured to nearest 0.01 degree Celsius) was recorded using HOBO (Onset Computer Corp., Pocasset, MA) environmental loggers (pre-set to record the water temperature every 15 min) positioned at the Lighthouse (c), Six Mile (f), Gap (g), Two Mile (h), San Sebastian (l) and Giants (r) acoustic receiver stations (Fig. 1).

### Statistical analysis

All statistical analysis were completed in R statistics version 4.0.3 “Bunnies-Wunnies Freak Out”^34^.

### Acoustic detections

Acoustic detections were imported into the GLATOS package of R statistics^37^. To remove false detections, VRL detection data were filtered using a time frame of 30× the tag nominal delay^36^. False detections were removed^38^, and detection data were reduced into discrete detection events under the assumption that if the same shark was not detected within the recommended^35^ time frame of 600 s, a new detection event would be logged when it was next detected. Two residency indices were used to assess sharks’ relative commitment to a receiver station. We used the Kessel method to divide the total distinct days each shark was detected by the number of distinct days it was detected at a specific location^39^, and the time interval method, (time interval used: 6 h) to divide the distinct number of times a shark was detected at each receiver location by the distinct number of time intervals in which a shark was detected at any receiver location^37^.

### Statistical analyses

*Carcharhinus leucas* and *C. limbatus* detection events were analysed using two generalised additive models (GAMs) in the mgcv package of R statistics (Table 2)^40–42^. Detection events were included as each model’s response variable, and seven measured parameters (receiver location, moon illumination, minute after the high tide, tidal range, water temperature, time of day and the day of the year) were included as potential explanatory terms. Each of the measured parameters were checked for cross-correlation before being included as a model response variable. The individual shark ID code (Table 1) was included as a random effect, and the effective receiver range (see: results) was included as an offset term. To pair shark sightings with the environmental data at a high resolution (recordings of temperature occurred every 15 min), receiver output files were converted to 15 min time slots with sharks present/absent^41^. A Tweedie distribution with a log link function was applied due to a high proportion of zeros in the data^43^. The receiver locations and individual tag codes were included as parametric terms^41^. Following AIC based model comparisons (Table 3), the tidal range was removed from both species models and the remaining five explanatory variables were smoothed with cubic splines. Cyclical cubic splines were applied to the day of the year, time from high tide and time of day^41^. Effective degrees of freedom (knots) were conservatively set to three for all parameters except for the day of the year whereby plots indicated six best mirrored inflections in plotted detection events. Models were summarized, plotted, and checked using the ‘gam.check’ function and assessed graphically by comparing the mean regression line with the 1:1 diagonal line^41,42^. Model checks did not reveal any obvious issues with convergence or overdispersion and did not indicate that per-variable *k* dimensions were low (< 0.5) (Supplementary Fig. 1).

**Table 3:** The final model parameters included in, and the deviance of, the most accurate model of *Carcharhinus limbatus* and *Carcharhinus leucas* detection events when considering the effects of the measured environmental and spatiotemporal parameters (receiver location, moon illumination, minute after the high tide, tidal range, water temperature, time of day and the day of the year). The tidal range was removed from the models of both species.

Genus species	Final model	Deviance explained (%)
*Carcharhinus leucas*	~ receiver location + moon illumination + minute after the high tide + water temperature + time of day + the day of the year	41.7
*Carcharhinus limbatus*	~ receiver location + moon illumination + minute after the high tide + water temperature + time of day + the day of the year	53.1

### Model selection

A stepwise backward selection procedure based on minimizing Akaike’s information criterion was applied to select the most accurate model of each species’ detection events^44^. Explanatory model parameters were sequentially deleted, and each new model iteration was compared with the last using a χ^2^ based analysis of variance (ANOVA) test under the assumption that a significantly different model (*p* < 0.05) with an improved deviance and lower Akaike’s information criterion indicated a more accurate fit for the model (Table 3).

## Supplementary Information

Supplementary Figure 1.

## Data Availability

All data is available upon reasonable request from the first author.
