# Supplementary material for: A Mozambican marine protected area provides important habitat for vulnerable pelagic sharks
Source: Sci Rep. 2023 Apr 20;13:6454. doi: 10.1038/s41598-023-32407-9 (PMC10119319; doi:10.1038/s41598-023-32407-9)
Supplement: Supplementary file 1 — Supplementary Figure 1. [file 41598_2023_32407_MOESM1_ESM.pdf]

A Mozambican marine protected area provides important habitat for vulnerable pelagic sharks

Calum J. G. Murie [1,2,*], Mario Lebrato [3], Andrew Lawrence[1], James Brown [1], Livia Gavard [2], Karen R. Bowles [3], Mauro G. Jije [3], Matt Dicken [4,5], Simon P. Oliver [1,6*]

[1] Department of Biological Sciences, University of Chester, Chester, CH1 4BJ, United Kingdom.

[2] The Underwater Africa Foundation, Tofo, Inhambane, Mozambique.

[3] Bazaruto Centre for Scientific Studies (BCSS), Bazaruto Archipelago, Inhambane, Mozambique.

[4] KwaZulu Natal Sharks Board, Umhlanga Rocks 4320, South Africa.

[5] School of Biological and Marine Sciences, University of Plymouth, Plymouth, PL4 8AA, UK.

[6] The Thresher Shark Research and Conservation Project, Malapascua Island, Cebu, The Philippines.

***Corresponding authors:**

calummurie@gmail.com

s.oliver@chester.ac.uk

Keywords: ecological monitoring; MPA; Carcharhinus; sharks; habitat use; coral reef; residency patterns; spatial ecology; acoustic telemetry; Indian Ocean.

Supplementary Figure

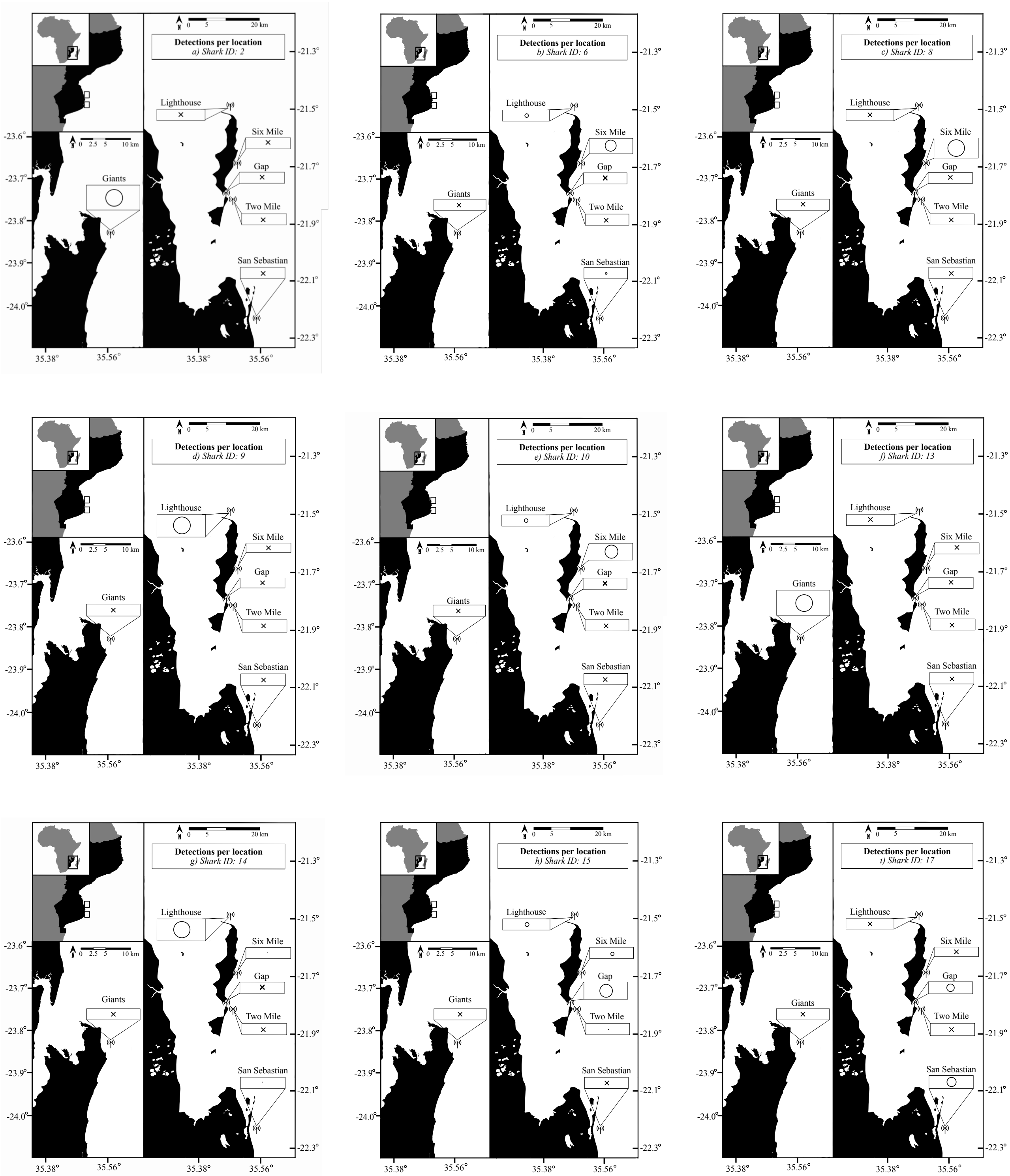

Supplementary Figure 1. The number of detections recorded for tagged bull, *Carcharhinus leucas* (a, b, c, d, e)*,* and oceanic blacktip, *Carcharhinus limbatus* (f, g, h, i)*,* sharks. The size of the circle describes the number of the detections that were recorded at that location. The radio antennas describe receiver locations that made at least one detection of a tagged shark. Shark ID number refer to table 1. An X denotes no detections of that shark at this site.
